# On the Job

**Published:** 1915-12

**Authors:** 


### On The Job.

We apologize for the colloquial expression, but there is much to be said in apology for it. There is a popular notion that professional men are irregular and not very energetic workers, and that the average business man works early and late, ruins his stomach with a hasty lunch and is on the job with scarcely any rest. During the last few years we have had occasion to see many business men, in many lines, and our experience is that there is just about one chance in three of finding anyone on the job at any given time during ordinary working hours; that most of them take from 12 or 12.30 to 2 for lunch; and that, with automobile trips in summer and numerous business trips in winter, the office grind is reduced to about five hours a day on the average. And we find not a few grown men taking a Saturday half holiday the year around without apology. We are even pessimistic enough to believe that the connection between an eight hour day and an 80-cent dollar (in purchasing power) is not entirely a coincidence.

The late Dr. Thomas F. Rochester used to paraphrase Benjamin Franklin's proverb thus: "Keep your office hour and your office hour will keep you." And we want to urge on the younger members of the profession that the keen competition of an over-crowded profession, necessitates close attention to duty rather than emulation of the habits of business men.

